# Uncovering the Role of Different Instructional Designs When Learning Tactical Scenes of Play through Dynamic Visualizations: A Systematic Review

**DOI:** 10.3390/ijerph18010256

**Published:** 2020-12-31

**Authors:** Ghazi Rekik, Yosra Belkhir, Mohamed Jarraya, Mohamed Amine Bouzid, Yung-Sheng Chen, Cheng-Deng Kuo

**Affiliations:** 1 Research Laboratory: Education, Motricity, Sport and Health (LR19JS01), High Institute of Sport and Physical Education, Sfax University, Sfax 3000, Tunisia; ghazi.rek@gmail.com (G.R.); belkhir.ysr@gmail.com (Y.B.); jarrayam@yahoo.fr (M.J.); bouzid.mohamed-amine@hotmail.fr (M.A.B.); 2 Al-Udhailiyah Primary School for Girls, Al-Farwaniyah 085700, Kuwait; 3 High Institute of Sport and Physical Education, Manouba University, Manouba 2010, Tunisia; 4 High Institute of Sport and Physical Education, Sfax University, Sfax 3000, Tunisia; 5 Department of Exercise and Health Sciences, University of Taipei, Taipei 111, Taiwan; 6 Exercise and Health Promotion Association, New Taipei City 241, Taiwan; 7 Department of Medical Research, Taipei Veterans General Hospital, Taipei 112, Taiwan; 8 Department of Internal Medicine, Taian Hospital, Taipei 104, Taiwan; 9 Tanyu Research Laboratory, Taipei 112, Taiwan

**Keywords:** cognitive load theory, dynamic visualizations, instructional designs, learning, team sports

## Abstract

Dynamic visualizations such as videos or animations have been developed to exchange information that transforms over time across a broad range of professional/academic contexts. However, such visual tools may impose substantial demands on the learner’s cognitive resources that are very limited in current knowledge. Cognitive load theory has been used to improve learning from dynamic visualizations by providing different instructional designs to manage learner cognitive load. This paper reviews a series of experimental studies assessing the effects of certain instructional designs on learning of tactical scenes of play through dynamic visualizations. An electronic database search was performed on the Web of Science and PubMed/Medline databases from inception to July 2020 using a combination of relevant keywords. Manual searches were also made. The search was limited to English language. A total of 515 records were screened by two researchers using the Population/Intervention/Comparison/Outcome(s) (PICO) criteria. The quality and validity of the included studies were assessed using “QualSyst”. Learning indicators in students and/or players (male and female) at any age category and competitive level were considered. Eleven studies met the inclusion criteria for this review, which focused on the effects of four instructional designs (i.e., using static visualizations, employing sequential presentation, applying segmentation, and decreasing presentation speed) on learning various game systems through dynamic visualizations. These studies indicate that (i) the effectiveness of all instructional designs depend upon the level of learners’ expertise when learning soccer/Australian football scenes through animations/videos, (ii) the effectiveness of using static visualizations instead of animations/videos showing soccer/basketball scenes depend upon the type of the depicted knowledge (i.e., motor knowledge or descriptive knowledge) for novice learners, (iii) the effectiveness of employing static visualizations and decreasing presentation speed when learning soccer/basketball scenes from animations/videos depend upon the level of content complexity, for novice learners. The current review demonstrated important practical implications for both coaches and physical education teachers using either animations and/or videos to communicate game systems. Indeed, findings suggested that adapting instructional designs to the level of learners’ expertise, type of depicted knowledge, and level of content complexity is a crucial part of effective tactical learning from dynamic visualizations.

## 1. Introduction

Dynamic visualizations are external representations that change over time and represent a non-stop flow of perceptual information, yielding an illusion of movements [1,2]. These pictorial demonstrations could be as animations used for communicating descriptive information/knowledge [3,4], or as realistic video clips used for portraying motor knowledge/skills [5,6]. The use of dynamic visualizations in learning environments can present numerous benefits. Firstly, they seem to be the most natural visual tool to convey dynamic properties (e.g., translation, transformation) that are tricky to describe verbally [7]. Secondly, they can depict dynamic information in an explicit and continuous way, which may help the observer to establish appropriate internal representation [8]. Thirdly, they can show the micro-steps of the dynamic phenomenon, while offering a concrete and global view [9] and avoiding the process of mental inference [10]. Fourthly, recent findings indicated that using dynamic visualizations in instructional contexts could be relevant for improving learners’ attitudes such as motivation and engagement [11,12,13]. In team-sport domain, dynamic presentation formats are expected to improve learners’ tactical knowledge by delivering directly visuo-spatial information (e.g., the players and the ball) and temporal, change-related information (e.g., the players’ movement) that helps the observer establish an appropriate internal representation of an ideal unfolding play [14,15]. Indeed, studying structured stimuli (i.e., scenes of organized playing patterns) is the obvious choice for learning team-ball game systems from dynamic visualizations, because unstructured stimuli break a play down with no apparent organization (e.g., scenes of players warming up, a break in play following an injury, etc.) [16].

Despite the presumed advantages of dynamic visualizations in learning, the Cognitive Load Theory (CLT) [17,18] argued that such visual tools may impose substantial demands for the learner’s cognitive resources that are very limited in both capacity and duration, which might hinder learning [19]. The CLT is a theory that considers how visual information impacts on Working Memory (WM) and learning. According to this theory, learning from dynamic visualizations depends specifically on two categories of cognitive load.

The first category is “the intrinsic cognitive load” which is dependent upon the levels of content complexity. From a cognitive load viewpoint, dealing with simple dynamic visualization (i.e., content with a little number of interactive elements) consumes less WM resources and leads to easier learning. In contrast, dealing with complex dynamic visualization (i.e., content with an excessive number of interactive elements) consumes large amounts of WM resources and makes learning process difficult [17]. In this framework, research within CLT [17,18] suggested two instructional designs, which effectively enable the control/management of the intrinsic cognitive load. The first technique is to employ “the sequential presentation method” [20]. This instructional strategy recommends presenting information depicted in dynamic visualization serially rather than concurrently. This method may be relevant for learning as it provides learners with less information to be concurrently treated in working memory and thus, facilitates the integration of information in long-term memory [21,22]. In addition, the sequential presentation of the dynamic visualizations’ components in a defined order could refer to a form of temporal cueing, facilitating the building of ordered knowledge in long-term memory [20]. The second technique is “the prediction method”. This strategy pushes learners to anticipate/predict future macro/micro steps of dynamic visualizations. This mental process is supposed to improve learning from dynamic representations as it encourages learners to activate their acquired knowledge of the system and/or help them to realize what they do not know about the system and stimulate a greater focus [10].

The second category is “the extraneous cognitive load” which is related to the designed instructional materials that interfere with schema acquisition. It is well known that the transient nature of information is responsible for the increase of extraneous cognitive load when learning from dynamic visualizations (the transient information effect) [18,23]. Indeed, videos or animations provide a transient, non-permanent stream of information that vanishes from the computer screen [17]. Consequently, learners are obliged to process current information while simultaneously trying to maintain the previously given information and integrate it with novel information in long-term memory [3,23,24]. To ovoid/reduce the transient information effect and improving learning from dynamic visualizations, research in the scope of CLT [17,18] suggested five instructional designs (without adding any oral/written explanations). The first technique is “the use of static visualizations” [2,25,26]. This method consists of replacing videos or animations with a series of static pictures or with a static diagram, describing the essential states of the dynamic system. This instructional strategy may decrease the extraneous cognitive load investment by allowing learners to benefit from sufficient time to identify and process relevant information and effectively integrate it in long-term memory [27,28]. Moreover, using static visualizations, compared to dynamic representations, offer the possibility to revise and compare different parts of the display as frequently as desired [29]. The second technique is “to employ segmentation” [30,31]. The segmentation of videos/animations corresponds to an insertion of pauses or time breaks between the key segments/steps of the dynamic phenomenon. This strategy provides learners with supplementary time to process and assimilate information received in the previous segments without having to simultaneously attend the next incoming information [31]. Moreover, this method could be referred to as temporal cueing because it allows learners to distinguish between macro/micro dynamic events in the display [32]. The third technique is “the incorporation of cues/signals” [33,34]. This instructional strategy can be applied by either “adding elements” such as arrows, lines, and thick frames, or “without adding elements” via coloring, flashing, and zooming [1]. According to the CLT, using cues or signals, especially without adding elements, in dynamic visualizations may improve learning because they are able to highlight the crucial information elements and thereby, to direct the learner’s attention towards it [35,36]. The fourth technique is “the decrease of presentation speed” [14,15]. This method consists of reducing the number of frames per second. Decreasing presentation speed of dynamic visualizations may provide learners with additional time to achieve the required cognitive processing in WM, while reducing the probability that key information is missing [37]. Moreover, such design technique is beneficial as it reduces the perceptual/cognitive demands by allowing learners to build a mental representation of local parts (i.e., micro/macro dynamic events), which then can be integrated into a coherent mental model [15,38]. The fifth technique is the use of learner-control [39,40,41]. This instructional design allows learners to control the dynamic display through interactive features such as stopping, replaying, reversing or changing speed. Using this method in computer-based learning environments allows learners to repeat and process the missed part of the display. Furthermore, this user-control give an additional time for learners to process, consolidate and transfer information into long-term memory before proceeding to the next segment/step [41].

With the growth in graphic technologies, dynamic visualizations such as realistic videos [42,43] or decorational animations [44,45] have been extensively employed by Physical Education (PE) teachers or coaches when teaching tactical knowledge in team-ball sports. However, as mentioned above, learning from dynamic visualizations could be a challenging task, because such visual tools may impose substantial demands on the learner’s cognitive resources that are very limited in current knowledge [18,23]. In this context, based on CLT, some past scientific works [2,13,15] have explored the effects of a variety of design techniques (without adding any oral/written explanations) on learning of tactical scenes from dynamic visualizations. Yet, research into the instructional and/or cognitive effects of these techniques has obtained mixed results. A synthesis of the literature related to the role of these instructional designs may be helpful and of great applicable relevance towards understanding how dynamic visualizations portraying game systems should be designed. This approach can help coaches and/or PE teachers to (i) determine the best dynamic format of visualization for an efficient learning of tactical scenes of play (ii) determine whether the effectiveness of specific instructional designs depend upon some moderator factors. Interestingly, a systematic review about this topic has not been published until today. Therefore, we attempted to fill this knowledge gap with the current paper by reviewing a series of experimental studies examining the potential effects of different instructional designs when learning game systems through dynamic visualizations.

## 2. Materials and Methods

### 2.1. Protocol

This paper reviews a series of statistical quantitative studies assessing the effects of different instructional designs on learning of tactical scenes of play through dynamic visualizations. This systematic review was conducted and reported in accordance with the preferred reporting items for systematic reviews and/or meta-analysis (PRISMA) guidelines [46].

### 2.2. Eligibility Criteria

To be suitable for inclusion, studies had to fulfill the following PICO criteria:

- Population: studies recruiting male and female students and/or players at any age category and competitive level as participants.
- Intervention or exposure: original investigations assessing the effects of instructional designs when learning tactical scenes of play through any type of dynamic visualization (i.e., video or animation)
- Outcome(s): studies involving cognitive load and/or learning measurements.
- Design: original investigations published in peer-reviewed journals.
- Time filter: Until 17 July 2020.
- Language filter: articles written in English language exclusively.

Studies not meeting with the above-mentioned PICO criteria were excluded, namely:

- Studies based on multimedia learning environment (i.e., combination of visual and oral/written explanations). This requirement was applied in order to avoid the occurrence of modality effect (for this point see [47,48]).
- Proceedings, case studies, encyclopedias, conference papers, thesis, reviews, book chapters, books, expert interviews, meta-analysis, or commentary articles. Overall, non-peer reviewed, or grey literature was discarded, in order to keep only high-quality studies.

### 2.3. Information Sources and Search

A preliminary literature search was conducted for available systematic review that had reported the role of instructional designs when learning tactical scenes of play through dynamic visualizations. No systematic reviews were found. Then, literature searches of the PubMed/Medline and Web of Science databases were performed without applying any time limits or filters; the final search being completed on 17 July 2020. The following combination of keywords was used: (dynamic visualization OR animation OR video) AND (segmentation OR static visualization OR pictures OR photographs OR sequential presentation OR learner control OR presentation speed OR signaling OR cuing OR prediction) AND (team sports OR soccer OR football OR basketball OR handball OR volleyball OR rugby OR futsal OR American football). Manual searches were also made using reference lists from the recovered articles in order to identify additional studies not included in these search terms. In addition, specific target journals (e.g., Journal of Computer Assisted Learning, Psychology of Sport and Exercise, Computers & Education, Computers in Human Behavior, Journal of Sports Sciences, Learning and Instruction, Applied Cognitive Psychology, British Journal of Educational Technology, Journal of Applied Sport Psychology, Educational Psychology Review, Journal of Sport and Exercise Psychology) were hand-searched for possible accepted studies in the field.

### 2.4. Study Selection

The initial database created from the two scholarly electronic databases was organized. Duplicate citations were removed by Endnote X8 and manually checked by the two first authors. Following the removal of duplicate studies from the two search databases, the researchers individually screened the articles by title and abstract to record the relevant studies. Selected papers were then read in full to finalize eligibility in accordance with the above-mentioned PICO criteria. Discordance was resolved by consensus. In case of uncertainty, discussion with the third co-author determined the final inclusion or exclusion of the article. The university’s library, electronic databases, and a search of personal files were used to obtain full copies of the published manuscripts.

### 2.5. Data Collection Process

The two first authors independently collected data using a pilot-tested extraction form, and they resolved any disagreements by consensus. In case of hesitation, conversation with the third co-author determined the final decision.

### 2.6. Data Items

Information was extracted from each study on: (1) type of instructional design, (2) authors and year of publication, (3) domain, (4) type of dynamic visualization, (5) type of depicted knowledge, (6) study sample, (7) dependent variables, and (8) key outcomes.

### 2.7. Risk of Bias in Individual Studies

The methodological quality and validity of each paper included in this systematic review were evaluated through the formal quantitative assessment tool “*QualSyst*” [49]. The QualSyst is a validated generic checklist and comprises 14 items scored in relation to the degree of meeting a specific criterion (yes = 2, partial = 1, no = 0), and it gives the possibility to score “not applicable” when an item is not applicable to a particular study. The testing procedure of subscale tests has been reported in Kmet et al. study’s [49] for the quantitative studies, with inter-rater agreement in scoring (by item) ranged from 73% to 100%. A percentage of quality was calculated for each article: [(total score across relevant items ÷ total possible score) × 100]. According to Trabelsi et al. [50], a percentage of ≥75% was considered as indicative of strong quality, a percentage of 55–75% as moderate quality, and a percentage of ≤55% as weak quality, when using the QualSyst assessment tool. Note that items judged “not applicable” were excluded from the calculation of the total score, and thus the maximum total possible score is 24 instead of 28. This process of coding was made, independently, by the two first authors. In case of disagreement, consensus was reached through discussion or consultation of the third co-author.

## 3. Results

### 3.1. Study Selection

**Figure 1:**
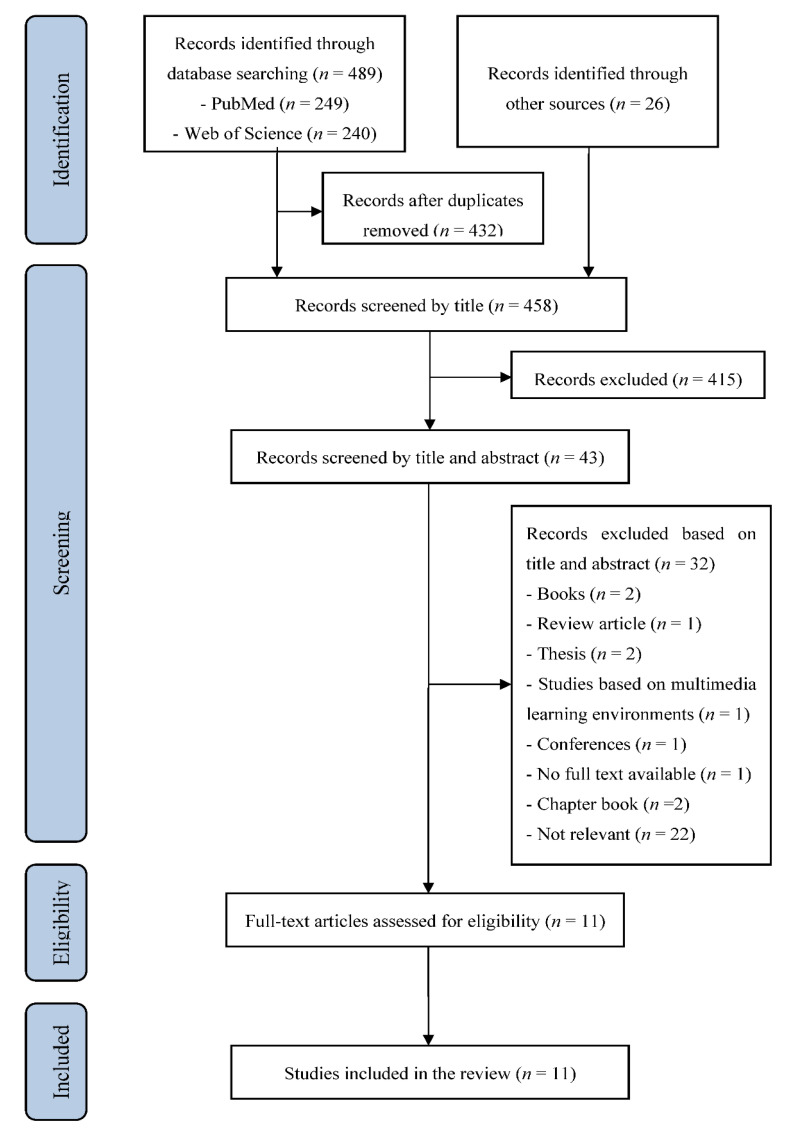
Flowchart illustrating the different phases of the search and study selection. A total of 11 articles fulfilled the eligibility criteria.

**Figure 2:**
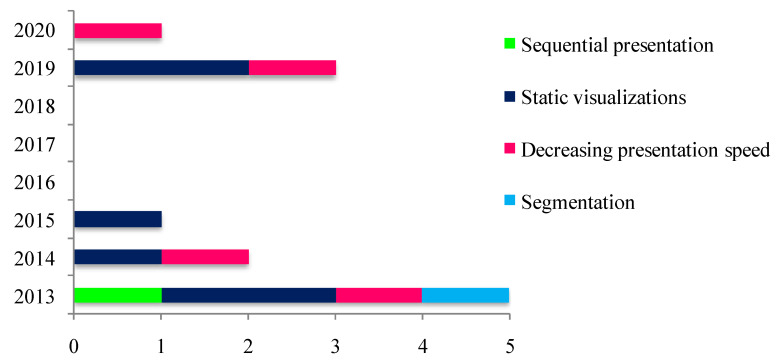
The distribution (by year and number) of studies focused on the role of instructional designs when learning tactical scenes of play through dynamic visualizations.

Electronic database searching yielded a preliminary pool of 489 possible records. 57 duplicate records were removed. Manual searches made using reference lists from the recovered records and specific targeted journals resulted in 26 additional records. Next, 458 records were examined. Subsequently, 415 records screened by title were excluded (not relevant), and 43 records were carefully screened by title and abstract. Afterward, 32 articles were excluded (2 books, 1 review article, 2 thesis, 1 study based on multimedia learning environment, 1 conference, 1 no full text available, 2 chapter books, 22 studies not assessing the effects of instructional designs). The remaining 11 articles were assessed for eligibility. They were eligible for inclusion in the current systematic review after a careful review of their full texts. The process used for selecting articles is outlined in Figure 1. These 11 papers were published between 2013 and 2020 in peer-reviewed journals (Figure 2).

### 3.2. Quality Assessment

The methodological quality and validity of each study included in our systematic review was deemed to be good to excellent, and no study was excluded because of low-quality scores: five studies receiving a score of 20 (83.33%), and six studies receiving a score of 21(87.5%) (Table 1).

### 3.3. Study Characteristics

The main characteristics of selected studies are given in Table 2. The included studies are focused, particularly, on the effects of four instructional designs when learning tactical scenes of play in basketball [2,13,51], soccer [14,15,20,26,30,52,53], and Australian football [54] through dynamic visualizations. One study [20] examined the effect of employing sequential presentation. Six studies [2,13,14,26,52,53] tested the effect of using static visualizations. Four studies explored the effect of decreasing presentation speed [14,15,51,54], and one study [30] examined the effect of using segmentation technique.

These investigations were conducted within physical education [2,13,51] or sports coaching [14,15,20,26,30,52,53,54] domains. Most of these studies were designed to evaluate the effects of these instructional designs on cognitive load, comprehension/recall accuracy (through a paper/pencil task), and/or game performance (during realistic situation) in order to obtain an indication of learning efficiency.

The participants of three studies [2,13,51] were novice students (males and females) recruited from Tunisian secondary school classes. They were aged between 15 and 16 years old. The participants of six studies [14,20,26,30,52,53] were either novice students (males) recruited from undergraduate French university classes (aged between 22 and 29 years old), or expert players (aged between 24 and 29 years old) engaged with French professional and/or semi-professional soccer clubs. The participants of one study [15] were sub-expert players (aged between 13 and 14 years old) engaged with teams from the second division of the Tunisian football league. The participants of one study [54] were novices (M_age_ = 22.68 years, SD = 4.05), sub-experts (M_age_ = 20.34 years, SD = 3.44) and experts (M_age_ = 22.19 years, SD = 3.10) Australian footballers (males).

### 3.4. Main Findings

Firstly, most of the reviewed studies [14,20,30,52,53,54] revealed that the effectiveness of the four identified instructional designs depend upon the level of learners’ expertise when learning soccer scenes from animations and Australian football scenes through realistic videos. Indeed, it was observed that using static visualizations, employing sequential presentation, using segmentation, and decreasing presentation speed are effective only for less knowledgeable learners (i.e., novices), but they become ineffective for more knowledgeable learners (i.e., experts).

Secondly, the present literature review showed that the effectiveness of using static visualizations, as instructional design, instead of dynamic visualizations showing tactical scenes depend upon the type of the depicted knowledge (i.e., motor knowledge or descriptive knowledge), particularly for novice learners. In fact, it has been observed that replacing animations portraying descriptive knowledge with a series of static pictures or diagrams induce positive effects when learning soccer scenes among less knowledgeable learners [14,26,52,53]. Conversely, using a series of static pictures instead of realistic videos portraying motor skills induce negative effects when learning basketball scenes among novice learners [2,13].

Thirdly, the review articles demonstrate that the effectiveness of two instructional designs (i.e., using static visualizations, and decreasing presentation speed) depend upon the level of content complexity, especially for novice learners. In this context, it has been established that replacing a soccer animation with an arrows-based diagram induce positive effects on learning complex soccer scene of play (i.e., with high content complexity), but negative effects on learning simple soccer scene of play (i.e., with low content complexity) [26]. Moreover, using a series of static pictures instead of realistic videos portraying motor skills in basketball induce similar effects on learning when the content complexity was low, and negative effects on learning when the content complexity was medium and/or high [13]. Furthermore, it was found that the instructional benefits of decreasing presentation speed of animations (showing descriptive knowledge in soccer) or realistic videos (showing motor skills in basketball) were present only when studying medium and/or high levels of content complexity [15,51].

Table 3 provides a summary of the suggested instructional designs in order to improve learning of tactical scenes of play through dynamic visualizations, as a function of these moderator factors.

## 4. Discussion

This paper reviews a series of experimental studies examining the effects of different instructional designs on learning of tactical scenes of play through dynamic visualizations. The literature search strategies yielded a final pool of eleven papers. These articles are focused to the effects of four instructional designs (using static visualizations, employing sequential presentation, applying segmentation, and decreasing presentation speed) when learning basketball, soccer, or Australian football game systems. Overall, research into the instructional and/or cognitive effects of these instructional designs has obtained mixed results. In fact, it has been observed that the effectiveness of these instructional designs when learning various tactical scenes of play from dynamic visualizations depends/varies as a function of three moderator factors: the level of learners’ expertise, type of depicted knowledge, and level of content complexity.

### 4.1. Level of Learners’ Expertise

The current state of the literature indicated that learner prior knowledge is a significant factor that could moderate the effectiveness of all identified instructional designs, when learning from animated soccer scenes (showing descriptive knowledge). Moreover, the effectiveness of decreasing presentation speed of realistic video showing Australian football was moderate by the level of learners’ expertise.

In this framework, Khacharem et al. [20] found that the effect of using sequential presentation was moderated by the level of players’ expertise when learning soccer drill from an animation. In this study, participants were invited to complete a recall-reconstruction test and to rate their invested mental effort after studying a concurrent or sequential presentation of a soccer animation. For novice players, the sequential presentation produced better learning outcomes. Conversely, expert players performed better after studying the concurrent presentation. Moreover, the effective use of the segmentation technique was also moderated by the level of learners’ expertise when studying complex soccer scenes from animations. Khacharem et al. [30] tested the effect of two types of segmentation (macro-step and micro-step) on learning soccer attacking drills. Even though results demonstrated positive effect of the macro-step segmentation among all players, novices benefited more from the micro-step segmentation than from the macro-step segmentation, while experts performed at the same level with both forms of segmentation. Furthermore, Khacharem et al. [52,53] investigated the effects of expertise on perceived cognitive load and performance resulting from studying soccer scene either through an animation or via a series of static pictures. The results showed that novice players achieved higher performance outcomes after studying static pictures. However, expert players performed better after studying instructional animations. Similarly, Khacharem et al. [14] found an interaction between levels of learner expertise and the usefulness of replacing an animation with a static picture when studying a soccer playing system. According to this study, displaying a static picture to novice players is more helpful for learning than displaying an animation. Conversely, learning from a continuous animation is more beneficial for expert players: they attained the higher level of performance with the same time on the immediate recall-test, needed lower number of repetitions, and invested less mental effort. Additionally, it was established that learners’ prior knowledge should be taken into consideration when decreasing speed of video and/or animation. For example, Khacharem et al. [14] showed that novice players achieved higher recall scores, needed a lower number of repetitions and invested less mental effort when the animations were played at a low speed than when they were played at a normal or high speed. However, expert players had to invest less mental effort to attain the same level of performance with the same number of repetitions, when the animations were displayed at a high or normal speed than when they were displayed at a low speed (see [54] for more similar results with video portraying motor skills in Australian football).

The interaction between the effectiveness of these instructional designs and levels of learners’ expertise when learning tactical scenes of play from dynamic visualizations is mainly due to “*the expertise reversal effect*” (for a review, see [55,56,57,58,59]. Accordingly, learning from dynamic visualizations depends not only on how the information is presented, but also on the quantity of the learner prior knowledge in the domain. It is well known that prior knowledge is stored in long-term memory as cognitive schemas, through experience and deliberate practice [53,60]. The development of domain-specific knowledge can effectively reduce WM overload by assembling a large amount of information elements into a single unit. As a result, experienced learners were able to deal with dynamic visualizations, by identifying the crucial aspects and ignore the unimportant ones [61,62]. Consequently, instructional designs that are optimal and effective for less knowledgeable learners may become ineffective and hinder learning for more knowledgeable learners, and vice versa [14,55,56].

### 4.2. Type of Depicted Knowledge

It has been observed that the type of knowledge (i.e., motor knowledge or descriptive knowledge) depicted in dynamic visualizations could moderate the effectiveness of one of the above-mentioned instructional designs (i.e., using static visualizations) when learning tactical scenes, only for novice learners.

On one hand, Khacharem and colleagues [52,53] found that replacing animations with a series of static pictures is an effective strategy for learning soccer attacking drills, especially for novice soccer players. Similarly, it was established that using a static picture representing three key stages of a soccer animation is more beneficial for learning: novice players attained the same level of performance with less time on the immediate recall-test, with lower number of repetitions, and with lower investment of mental effort [14]. As mentioned in the introduction, using static instead of dynamic visualizations, especially for novices, may decrease the extraneous cognitive load investment by allowing learners to benefit from sufficient time to identify and process relevant information and effectively integrate it in long-term memory [27,28]. Moreover, using static visualizations, compared to dynamic representations, offer the possibility to revise and compare different parts of the display as frequently as desired [29].

One the other hand, evidence of positive effects of using static visualizations were not proved in comparison with using dynamic visualizations among novice learners, when it was about learning motor knowledge/skills. In this context, Rekik et al. [2] explored the effectiveness of realistic video versus a series of static photographs on learning basketball tactical actions within physical education domain. Immediately after the learning phase, students were asked to indicate their cognitive load investment. Next, they were invited to perform a game understanding task and a game performance task. For all indicators, the results showed that learning from the video was more effective than learning from a series of photographs. These results are consistent with previous research carried out in non-sporting domains, demonstrating the cognitive and instructional value of dynamic visualizations (as opposed to statics) involving various motor skills that require hand manipulations such as performing an emergency procedure [9], making origami shapes [63], and tying diverse knots [64,65]. Following the neuroscience literature, the superiority of dynamic visualizations over statics when learning motor knowledge/skills is mainly due to the activation of the Mirror-Neuron System [66,67,68,69]. This system was originally identified in primates. It is a neuro-physiological circuit distributed across the pre-motor cortex that is automatically activated when someone is observing another person performing an action [67,69]. Moreover, as humans’ actions are part of primary knowledge such as face recognition, learning from others, and language, their acquisition is very easy and requires little cognitive effort [70]. Hence, watching dynamic visualizations involving motor skills does not require excessive cognitive resources, because humans are biologically evolved to effectively acquire such kind of knowledge. The phenomenon of learning motor skills from dynamic visualizations compared to statics was called “the human movement effect” [70].

### 4.3. Level of Content Complexity

Analysis of the selected articles showed that the level of content complexity (i.e., the number of interactive information elements) is a significant factor that could modulate the effectiveness of two instructional designs (i.e., using static visualizations, decreasing presentation speed) when learning tactical game systems through dynamic visualizations (animations and videos), solely for novice learners. The term “*complexity*” used in these experimental studies referred to the internal complexity of the playing systems that was associated with the intrinsic cognitive load [71]. In fact, the more complex scene of play is the situation that involves more players and more interactions between them [72,73].

In this framework, Khacharem et al. [26] showed that replacing an animation with an arrows-based diagram was efficacious only when studying complex soccer scene of play (i.e., with high content complexity). Indeed, novice players achieved the same level of comprehension with lower investment of mental effort. By contrast, participants learned more efficiently from the animation than from the static diagram when it is about a simple soccer scene. In the same vein, Rekik et al. [13] found that using a series of static pictures or a video had similar effects among novice participants when learning basketball scenes with low content complexity. However, for medium and high content complexity, the dynamic format had a clear advantage over the static format in terms of cognitive load investment and learning outcomes. In addition, it was found that the instructional benefits of decreasing presentation speed of animations showing descriptive knowledge or videos showing motor skills were also affected by the level of content complexity. In this context, Rekik et al. [15] examined the effect of content complexity on learning from soccer animations presented either at normal or low speeds (i.e., 0.5- and 1.0-times normal speed). The results revealed that while the decrease of presentation speed had no advantages when learning low-complexity content, sub-expert players profited more from the low than the normal presentation speed when learning high complexity content (based on the combination of comprehension and cognitive load scores). The same pattern of results was obtained when learning basketball tactical actions through videos modeling examples [51]. Authors found that both speeds of presentation have similar effects when learning low content complexity. Conversely, for medium and high complexity contents, novice participants exposed to the slow-presentation speed learned more efficiently than those exposed to the normal-presentation speed.

These researchers referred usually to the CLT [17,18] in order to explain the interaction between the effectiveness of instructional designs and the levels of content complexity when learning game systems from dynamic visualizations. Indeed, dynamic formats displaying contents with low levels of complexity led to easier learning, because learners had to consume less perceptual-cognitive resources to deal with both the transient nature of information and few numbers of interactive information elements. As a result, learners were not forced to integrate and maintain excessive information elements in WM. Consequently, novice learners could benefit from videos or animations showing tactical scenes of play without running the risk of a potential cognitive overload. By contrast, dealing with more complex dynamic visualizations made learning difficult and consumed a large amount of perceptual-cognitive resources, as learners were asked to deal with the transient nature of information and to spatially split their attention among the excessive number of interactive elements [17,18]. Therefore, the use of the above-mentioned instructional designs (except the use of static visualizations when learning motor skills; due to the human movement effect) might reduce these cognitive processing demands and improve novices’ performance when learning tactical scenes of play through dynamic visualizations.

### 4.4. Strengths and Weaknesses

The strengths of the present study include a comprehensive coverage of the available literature and the careful appraisal of its quality, via the utilization of a wide range of key words (related to the relationships between dynamic visualizations, instructional designs, and team sports) searched through two globe databases, and the high methodological quality of the included studies. However, despite its novelty, certain limitations should be kept in mind when interpreting results. First, the electronic database search was performed solely on the Web of Science and PubMed/MEDLINE. Further studies are required to enlarge the sample by including other electronic databases such as SCOPUS, ERIC, and PsycINFO. Second, the number of good quality studies evaluating the effects of instructional designs on learning tactical scenes from dynamic visualizations is limited, hampering the ability to draw definitive conclusions. Third, most of the included studies were focused on short-term learning, because the indicators of learning were collected immediately after students/players had viewed the instructional materials. Fourth, all reviewed papers do not take into consideration the gender of learners. Fifth, most of the included studies (except Rekik et al. [2,13]) are low in ecological validity as the learning measurements were performed under laboratory conditions. Lastly, the present literature review was interested solely on learning of playing systems. In fact, dynamic visualizations have been extensively employed by PE teachers and/or coaches to improve learning of technical skills [74,75,76,77]. Thus, more systematic reviews are required to uncover the role of instructional designs when learning from dynamic visualizations portraying actions/events in individual sports.

## 5. Conclusions

The current review demonstrated important practical implications for both coaches and PE teachers using either animations or realistic video clips to communicate/explain different playing systems. It offers insight into the effectiveness of a variety of instructional designs (using static visualizations, employing sequential presentation, applying segmentation, and decreasing presentation speed) when learning about tactical scenes of play through dynamic visualizations. Overall, research into the instructional and/or cognitive effects of these instructional designs has obtained mixed results. Indeed, the findings suggested that adapting various instructional designs to the level of learners’ expertise, type of depicted knowledge, and level of content complexity is a crucial part of effective tactical learning from dynamic visualizations.

## Figures and Tables

**Table 1:** Quality appraisal of included studies.

Study	Question Described	Appropriate Study Design	Appropriate Subject Selection	Subjects’ Characteristics Described	Random Allocation	Researchers Blinded	Subjects Blinded	Outcome Measures Well Defined and Robust to Bias	Sample Size Appropriate	Analytic Methods Well Described	Estimate of Variance Reported	Controlled for Confounding	Results Reported in Detail	Conclusion Supported by Results	Total Score	Quality (%)
Khacharem et al. [30]	2	2	1	1	2	NA	NA	1	1	2	2	2	2	2	20	83.33%
Khacharem et al. [20]	2	2	2	2	1	NA	NA	1	1	2	2	2	2	2	21	87.5%
Rekik et al. [2]	2	2	1	1	2	NA	NA	2	2	1	2	2	2	2	21	87.5%
Lorains et al. [54]	2	2	2	2	2	NA	NA	1	2	1	2	2	1	1	20	83.33%
Khacharem et al. [26]	2	2	1	2	2	NA	NA	1	1	2	2	2	2	2	21	87.5%
Rekik et al. [13]	2	2	1	2	2	NA	NA	2	1	1	2	2	2	2	21	87.5%
Khacharem et al. [52]	2	2	2	2	1	NA	NA	1	1	2	2	2	1	2	20	83.33%
Jarraya et al. [51]	2	2	1	2	2	NA	NA	2	1	1	2	2	2	2	21	87.5%
Khacharem et al. [14]	2	2	2	2	1	NA	NA	1	1	2	2	2	1	2	20	83.33%
Khacharem et al. [35]	2	2	2	2	1	NA	NA	1	2	2	2	2	2	1	21	87.5%
Rekik et al. [15]	2	2	1	2	2	NA	NA	1	1	2	2	2	1	2	20	83.33%

NA = not applicated.

**Table 2:** Effects of instructional designs on learning tactical scenes of play through dynamic visualizations: overview of the analyzed papers.

Instructional Designs	Source	Domain	Dynamic Visualization	Depicted Knowledge	Sample	Dependent Variables	Key Outcomes
Sequential presentation	Khacharem et al. [20]	Soccer	Animation	Descriptive	Novices Experts	Recall accuracy	*For Novices*: Sequential > concurrent *For experts*: Sequential = concurrent
						Mental Effort	*For Novices*: Sequential < concurrent *For experts*: Sequential > concurrent
						Number of repetition	*For Novices*: Sequential = concurrent *For experts*: Sequential = concurrent
						Learning Efficiency	*For Novices*: Sequential > concurrent *For experts*: Sequential < concurrent
Static visualizations	Rekik et al. [2]	Basketball	Video	Motor skills	Novices	Cognitive load Comprehension Game performance	Video < Series of pictures Video > Series of pictures Video > Series of pictures
	Khacharem et al. [52]	Soccer	Animation	Descriptive	Novices Experts	Mental Effort	*For Novices*: Series of pictures > Animation > Combined *For Experts*: Animation < Series of pictures < Combined
						Recall-Performance	*For Novices*: Animation = Series of pictures < Combined *For Experts*: Animation > Series of pictures > Combined
						Number of repetitions	*For Novices*: Series of pictures > Animation > Combined *For Experts*: Animation < Series of pictures < Combined
						Learning Efficiency	*For Novices*: Series of pictures > Animation > Combined *For Experts*: Animation > Series of pictures > Combined
	Khacharem et al. [53]	Soccer	Animation	Descriptive	Novices Experts	Recall accuracy	*For Novices*: Animation < Series of pictures without tracing < Series of pictures with tracing *For experts*: Animation = Series of pictures without tracing = Series of pictures with tracing
						Mental Effort	*For Novices*: Series of pictures with tracing < Animation = Series of pictures without tracing *For experts*: Animation < Series of pictures without tracing = Series of pictures with tracing
						Number of Repetitions	*For Novices*: Series of pictures with tracing < Animation = Series of pictures without tracing *For experts*: Animation = Series of pictures without tracing = Series of pictures with tracing
						Learning Efficiency	*For Novices*: Animation < Series of pictures without tracing < Series of pictures with tracing *For experts*: Animation > Series of pictures without tracing = Series of pictures with tracing
	Rekik et al. [13]	Basketball	Video	Motor skills	Novices	Cognitive load	*For low content complexity*: Video = Series of pictures *For medium/high contents complexity*: Video < Series of pictures
						Comprehension	*For low content complexity*: Video = Series of pictures *For medium/high contents complexity*: Video > Series of pictures
						Game performance	*For low content complexity*: Video = Series of pictures *For medium/high contents complexity*: Video > Series of pictures
	Khacharem et al. [14]	Soccer	Animation	Descriptive	Novices Experts	Recall accuracy	*For Novices*: Animation = Picture *For Experts*: Animation > Picture
						Time on immediate recall test	*For Novices*: Animation > Picture *For Experts*: Animation = Picture
						Mental Effort	*For Novices*: Animation > Picture *For Experts*: Animation < Picture
						Number of repetitions	*For Novices*: Animation > Picture *For Experts*: Animation < Picture
						Learning Efficiency	*For Novices*: Animation < Picture *For Experts*: Animation > Picture
						Delayed recall accuracy	*For Novices*: Animation < Picture *For Experts*: Animation > Picture
						Time on delayed recall test	*For Novices*: Animation > Picture *For Experts*: Animation = Picture
	Khacharem et al. [26]	Soccer	Animation	Descriptive	Novices	Performance	*For low content complexity*: Animation = diagram *For high content complexity*: Animation < diagram
						Mental Effort	*For low content complexity*: Animation < diagram *For high content complexity*: Animation = diagram
						Learning Efficiency	*For low content complexity*: Animation > diagram *For high content complexity*: Animation < diagram
Decreasing presentation speed	Lorains et al. [54]	Australian football	Video	Motor skills	Novices Sub-Experts Experts	Decision accuracy	*For Novices and Sub-Experts*: low speed = Normal speed < high speeds *For Experts*: high speeds > Normal speed = low speed
	Jarraya et al. [51]	Basketball	Video	Motor skills	Novices	Mental Effort	*For low content complexity*: Normal speed = low speed *For medium/high contents complexity*: Normal speed < low speed
						Game performance	*For low content complexity*: Normal speed = low speed *For medium/high contents complexity*: Normal speed < low speed
						Learning Efficiency	*For low content complexity*: Normal speed = low speed *For high content complexity*: Normal speed < low speed
	Khacharem et al. [14]	Soccer	Animation	Descriptive	Novices Experts	Recall accuracy	*For Novices*: High speed = Normal speed < low speed *For Experts*: High speed = Normal speed = low speed
						Time on immediate recall test	*For Novices*: High speed > Normal speed > low speed *For Experts*: High speed < low speed = Normal speed
						Mental Effort	*For Novices*: High speed > Normal speed > low speed *For Experts*: High speed = Normal speed < low speed
						Number of repetitions	*For Novices*: High speed > Normal speed > low speed *For Experts*: High speed = Normal speed = low speed
						Learning Efficiency	*For Novices*: High speed < Normal speed < low speed *For Experts*: High speed = Normal speed > low speed
						Delayed recall accuracy	*For Novices*: High speed = Normal speed < low speed *For Experts*: High speed = Normal speed = low speed
						Time on delayed recall test	*For Novices*: High speed = Normal speed < low speed *For Experts*: High speed < Normal speed = low speed
	Rekik et al. [15]	Soccer	Animation	Descriptive	Sub-Experts	Mental Effort	*For low content complexity*: Normal speed = low speed *For high content complexity*: Normal speed > low speed
						Comprehension	*For low content complexity*: Normal speed = low speed *For high content complexity*: Normal speed < low speed
						Learning Efficiency	*For low content complexity*: Normal speed = low speed *For high content complexity*: Normal speed < low speed
Segmentation	Khacharem et al. [30]	Soccer	Animation	Descriptive	Novices Experts	Recall accuracy	*For Novices*: Continuous = Macro-step = Micro-step *For experts*: Continuous < Macro-step < Micro-step
						Mental Effort	*For Novices*: Continuous > Macro-step > Micro-step *For experts*: Continuous > Macro-step = Micro-step
						Number of repetition	*For Novices*: Continuous > Macro-step > Micro-step *For experts*: Continuous > Macro-step = Micro-step
						Learning Efficiency	*For Novices*: Continuous < Macro-step < Micro-step *For experts*: Continuous < Macro-step = Micro-step

**Table 3:** Suggested instructional designs to improve learning of tactical scenes of play through dynamic visualizations.

Dynamic Visualization	Depicted Knowledge	Level of Content Complexity	Suggested Design Technique	Addressed to	Reference
Animation	Descriptive	High	Sequential presentation	Novices	[20]
			Static visualizations	Novices	[14,26,52,53]
			Decreasing presentation speed	Novices/sub-Experts	[14,15]
			Segmentation (Micro-step)	Novices	[30]
			Segmentation (Macro-step)	Novices/Experts	[30]
Video	Motor skills	Medium/High	Decreasing presentation speed	Novices	[51]

## Data Availability

The data presented in this study are available on request from the corresponding author.
